# Myocardial sympathetic distal axon loss in subjects with Lewy pathology: an artificial intelligence‐based histopathological analysis

**DOI:** 10.1002/alz70861_108548

**Published:** 2025-12-23

**Authors:** Ville Kivistö, Benjamin Englert, Jarno Tuimala, Eloise H Kok, Henri Puttonen, Anna Raunio, Pekka Karhunen, Maria K. Lehtinen, Per Borghammer, Ella Ahvenainen, Kia Colangelo, Sara Savola, Maarit Tanskanen, Karri Kaivola, Pentti J. Tienari, Darshan Kumar, Anders Paetau, Olli Tynninen, Mikko I. Mäyränpää, Tuomo Polvikoski, Liisa Myllykangas

**Affiliations:** ^1^ University of Helsinki, Helsinki Finland; ^2^ HUS Diagnostic Center, Helsinki Finland; ^3^ Tampere University, Tampere, Pirkanmaa Finland; ^4^ Tampere University, Tampere Finland; ^5^ Fimlab Laboratories, Tampere Finland; ^6^ Boston Children's Hospital, Boston, MA USA; ^7^ Harvard Medical School, Boston, MA USA; ^8^ Insitute of Clinical Medicine, Aarhus Denmark; ^9^ Helsinki University Hospital, Helsinki Finland; ^10^ Aiforia Technologies Plc., Helsinki, Uusimaa Finland; ^11^ Newcastle University, Newcastle upon Tyne UK

## Abstract

**Background:**

Cardiac manifestations, including arrhythmias and sudden deaths, are associated with Lewy body disease (LBD), but studies addressing the underlying histopathological mechanisms at the myocardial level are sparse. Increasing evidence supports the existence of body‐first and brain‐first subtypes of LBD, whose effects on cardiac sympathetic innervation have not been studied at the histopathological level.

**Method:**

We generated an artificial intelligence‐based image recognition algorithm to quantify tyrosine hydroxylase (TH)‐immunoreactive sympathetic distal axons at the myocardial level. This novel tool was applied to septal samples of the population‐based Vantaa 85+ Study (*n* =138), and left ventricle samples of the Helsinki Biobank (*n* =87) and the forensic Tampere Sudden Death Study (TSDS, n=127). The level of myocardial TH‐reactivity was compared between subjects with and without Lewy pathology in the central nervous system in all cohorts. In the Vantaa 85+ Study, TH‐reactivity was also compared between subjects with body‐first and brain‐first subtypes, and potential confounding factors (age at death, sex, myocardial infarction, senile systemic amyloidosis, and diabetes medication) were controlled for using multiple linear regression models. In addition, left ventricle samples of the Helsinki Biobank and TSDS cohorts were immunohistochemically stained with an α‐synuclein antibody and semiquantitatively scored, and the results compared with levels of myocardial TH‐reactivity.

**Result:**

Presence of Lewy pathology was strongly associated with loss of sympathetic distal axons at the myocardial level in all three autopsy cohorts (p ≤ 0.001). In the Vantaa 85+ Study, the body‐first subtype (p < 0.001), but not the brain‐first subtype (p = 0.60), was associated with loss of sympathetic distal axons, and this association was independent of other known causes of sympathetic denervation. The body‐first subtype and myocardial infarction were the strongest predictors of myocardial sympathetic denervation in the oldest‐old population (Vantaa 85+). In accordance with previous literature, subjects with sparse or moderate α‐synuclein pathology in the heart showed the most severe loss of myocardial TH‐reactivity (Helsinki Biobank and TSDS).

**Conclusion:**

Lewy pathology in the central nervous system is strongly associated with loss of sympathetic distal axons at the myocardial level. The body‐first LBD subtype is one of the strongest predictors of myocardial sympathetic denervation in the oldest‐old population.

